# Multinational validation of the PREVENT and SCORE2 cardiovascular risk equations across 6.4 million individuals

**DOI:** 10.1038/s41591-026-04437-z

**Published:** 2026-05-05

**Authors:** Brendon L. Neuen, Rupert W. Major, Morgan E. Grams, Yingying Sang, Josef Coresh, Shoshana H. Ballew, Aditya Surapaneni, Natalia Alencar de Pinho, Johan Ärnlöv, Clare Arnott, Samira Bell, Jarett Berry, Hermann Brenner, Elizabeth Ciemins, Alexander R. Chang, Mary Cushman, James A. de Lemos, Juan Miguel Diaz-Tocados, Alfredo E. Farjat, Robert A. Fletcher, Ron T. Gansevoort, Hiddo J. L. Heerspink, William G. Herrington, Chuan Hong, Edward J. Horwitz, Shih-Jen Hwang, Simerjot K. Jassal, Philip A. Kalra, Ronit Katz, Sadiya S. Khan, Csaba P. Kovesdy, Florian Kronenberg, Jennifer S. Lees, Donald M. Lloyd-Jones, Jose Geraldo Mill, David M. J. Naimark, Chiadi Ndumele, Kevan R. Polkinghorne, Bruce M. Psaty, Patrick Schloemer, Michael G. Shlipak, Prabin Shrestha, Zean Song, Natalie Staplin, Dominik Steubl, Priya Vart, Frank L. J. Visseren, Mark Woodward, Kazumasa Yamagishi, Bessie A. Young, Charumathi Sabanayagam, Jose M. Valdivielso, CKD Prognosis Consortium investigators, Mark Woodward, John Chalmers, Katie Harris, Zean Song, Hiroshi Yatsuya, Yuanying Li, Midori Takada, Hiddo J. L. Heerspink, Priya Vart, Kevan R. Polkinghorne, Steven Chadban, Brendon L. Neuen, Robert A. Fletcher, Bruce Neal, Dominik Steubl, Dominik Steubl, Kazumasa Yamagishi, Isao Muraki, Hironori Imano, Hiroyasu Iso, Natalia Alencar de Pinho, Ziad Massy, Benedicte Stengel, Marie Metzger, Clare Arnott, Kenneth W. Mahaffey, Meg Jardine, Vlado Perkovic, Matthew Weir, Jing Chen, James Lash, Panduranga Rao, Hiddo J. L. Heerspink, Priya Vart, Colby Ayers, Amit Khera, Parag Joshi, Amil Shah, Michael J. Pencina, Matthew Engelhard, Mu Niu, Haoyuan Wang, Paula Bracco, Maria de Jesus Mendes da Fonseca, Isabela Bensenor, Maria da Conceição Chagas de Almeida, Luana Giatti, William G. Herrington, Richard Haynes, Parminder Judge, Alfredo E. Farjat, Patrick Schloemer, Ulla T. Schultheiss, Wibke Bechtel-Walz, Markus Schneider, Turgay Saritas, Ewan Pearson, Colin Palmer, Shona J. Livingstone, Emilie Lambourg, Serafi Cambray, Marcelino Bermudez-Lopez, Maria Luisa Martin-Conde, Maite Caus, Elizabeth Ciemins, Dominik Steubl, Rupert W. Major, Nigel J. Brunskill, James F. Medcalf, Simerjot K. Jassal, Jaclyn Bergstrom, Joachim Ix, Dena Rifkin, Csaba P. Kovesdy, Prabin Shrestha, Keiichi Sumida, Mary Cushman, Orlando Gutierrez, Katharine Cheung, Titi Ilori, Hiddo J. L. Heerspink, Priya Vart, Ching Yu Cheng, Cancan Xue, Xiayin Zhang, Crystal Chong, Natalie Staplin, Richard Haynes, Colin Baigent, Martin Landray, Rajkumar Chinnadurai, Smeeta Sinha, Sharmilee Rengarajan, Ivona Baricevic-Jones, Frank L. J. Visseren, Robin W. M. Vernooij, Hiddo J. L. Heerspink, Priya Vart, Simon Benjamin Ascher, David M. J. Naimark, Navdeep Tangri, Dominik Steubl, Anders Larsson, Vilmantas Giedraitis

**Affiliations:** 1 The George Institute for Global Health, University of New South Wales, Sydney, New South Wales Australia; 2 Department of Renal Medicine, Royal North Shore Hospital, Sydney, New South Wales Australia; 3 School of Medical Sciences – Public Health and Epidemiology Division (SAPPHIRE Group) College of Life Sciences, University of Leicester, Leicester, UK; 4 NIHR Leicester Biomedical Research Centre, Leicester, UK; 5 University Hospitals of Leicester NHS Trust, Leicester, UK; 6 Leicester, Leicestershire and Rutland Integrated Care Board, Leicester, UK; 7 Division of Precision Medicine, Department of Medicine, New York University Grossman School of Medicine, New York, NY USA; 8 Optimal Aging Institute, Department of Population Health, New York University Grossman School of Medicine, New York, NY USA; 9 Centre for Research in Epidemiology and Population Health (CESP), Clinical Epidemiology and Pharmacoepidemiology Team, Inserm U1018, Paris-Saclay University, Villejuif, France; 10 Department of Neurobiology, Care Sciences and Society, Division of Family Medicine and Primary Care, Karolinska Institute, Stockholm, Sweden; 11 Department of Cardiology, St. Vincent’s Hospital, Sydney, New South Wales Australia; 12 Department of Cardiology, Royal Prince Alfred Hospital, Sydney, New South Wales Australia; 13 Division of Population Health and Genomics, School of Medicine, University of Dundee, Dundee, UK; 14 Division of Population and Behavioural Sciences, School of Medicine, University of St Andrews, St Andrews, UK; 15 Department of Medicine, The University of Texas at Tyler School of Medicine, Tyler, TX USA; 16 Cancer Prevention Graduate School, German Cancer Research Center (DKFZ), Heidelberg, Germany; 17 Network Aging Research (NAR), Heidelberg University, Heidelberg, Germany; 18 American Medical Group Association, Alexandria, VA USA; 19 Department of Nephrology, Geisinger Medical Center, Danville, PA USA; 20 Department of Medicine, Larner College of Medicine at the University of Vermont, Burlington, VT USA; 21 Division of Cardiology, Department of Internal Medicine, The University of Texas Southwestern Medical Center, Dallas, TX USA; 22 Vascular and Renal Translational Research Group, Biomedical Research Institute of Lleida, Dr. Pifarré Foundation (IRBLleida), RICORS 2024, Lleida, Spain; 23 Clinical Statistics & Analytics, Bayer BV, Hoofddorp, The Netherlands; 24 British Heart Foundation Cardiovascular Epidemiology Unit, Department of Public Health and Primary Care, University of Cambridge, Cambridge, UK; 25 Victor Phillip Dahdaleh Heart and Lung Research Institute, University of Cambridge, Cambridge, UK; 26 University Medical Centre Groningen, Groningen, The Netherlands; 27 Department of Clinical Pharmacy and Pharmacology, University of Groningen, Groningen, The Netherlands; 28 Renal Studies Group, Clinical Trial Service Unit and Epidemiological Studies Unit (CTSU), Nuffield Department of Population Health, University of Oxford, Oxford, UK; 29 Oxford Kidney Unit, Oxford University Hospitals NHS Foundation Trust, Oxford, UK; 30 Department of Biostatistics & Bioinformatics, Duke University School of Medicine, Durham, NC USA; 31 Department of Medicine, Division of Nephrology & Hypertension, MetroHealth Medical Center, Case Western Reserve University School of Medicine, Cleveland, OH USA; 32 Framingham Heart Study, Framingham, MA USA; 33 Population Sciences Branch, National Heart, Lung and Blood Institute, National Institutes of Health, Bethesda, MD USA; 34 VA San Diego Healthcare System and Division of General Internal Medicine, Department of Medicine, University of California, San Diego, San Diego, CA USA; 35 VA San Diego Healthcare System, San Diego, CA USA; 36 Salford Royal Hospital, Northern Care Alliance NHS Foundation Trust, Salford, UK; 37 Department of Obstetrics and Gynecology, University of Washington, Seattle, WA USA; 38 Department of Medicine, Northwestern University Feinberg School of Medicine, Chicago, IL USA; 39 The University of Tennessee Health Science Center College of Medicine, Memphis, TN USA; 40 Memphis VA Medical Center, Memphis, TN USA; 41 Department of Genetics, Institute of Genetic Epidemiology, Medical University of Innsbruck, Innsbruck, Austria; 42 School of Cardiovascular and Metabolic Health, University of Glasgow, Glasgow, UK; 43 Framingham Center for Population and Prevention Science and Department of Medicine, Boston University Chobanian & Avedisian School of Medicine, Boston, MA USA; 44 Department of Physiological Sciences and Cassiano Antonio Moraes University Hospital (Hucam/Ebserh), Federal University of Espírito Santo, Vitória, Brazil; 45 Faculty of Medicine, University of Toronto, Toronto, Ontario Canada; 46 Institute of Health Policy, Management and Evaluation, Dalla Lana School of Public Health, University of Toronto, Toronto, Ontario Canada; 47 Sunnybrook Health Sciences Centre, Toronto, Ontario Canada; 48 Johns Hopkins Ciccarone Center for the Prevention of Cardiovascular Disease, Baltimore, MD USA; 49 Welch Center for Prevention, Epidemiology, and Clinical Research, Johns Hopkins University, Baltimore, MD USA; 50 Department of Nephrology, Monash Medical Centre, Monash Health, Melbourne, Victoria Australia; 51 Department of Medicine, Monash University, Melbourne, Victoria Australia; 52 School of Public Health and Preventive Medicine, Monash University, Melbourne, Victoria Australia; 53 Cardiovascular Health Research Unit, Departments of Medicine and Epidemiology, University of Washington, Seattle, WA USA; 54 Clinical Statistics & Analytics, Pharmaceuticals, Bayer AG, Berlin, Germany; 55 Kidney Health Research Collaborative, San Francisco Veterans Affairs Health Care System, University of California, San Francisco, San Francisco, CA USA; 56 Department of Public Health and Health Systems, Nagoya University Graduate School of Medicine, Nagoya, Japan; 57 Boehringer Ingelheim International GmbH, Ingelheim, Germany; 58 Department of Nephrology, Hospital rechts der Isar, Technical University Munich, Munich, Germany; 59 Department of Vascular Medicine, Utrecht University, Utrecht, The Netherlands; 60 The George Institute for Global Health, School of Public Health, Imperial College London, London, UK; 61 Department of Public Health Medicine, Institute of Medicine, and Health Services Research and Development Center, University of Tsukuba, Tsukuba, Japan; 62 Department of Public Health, Graduate School of Medicine, Juntendo University, Tokyo, Japan; 63 UW JEDI Center for Transformational Research, Office of Healthcare Equity, and Division of Nephrology, Department of Medicine, University of Washington, Seattle, WA USA; 64 Singapore Eye Research Institute, Singapore National Eye Centre, Singapore, Singapore and Duke-NUS Medical School, Singapore, Singapore; 65 Kidney Node, Charles Perkins Centre, University of Sydney, Sydney, New South Wales Australia; 66 Department of Renal Medicine, Kidney Centre, Royal Prince Alfred Hospital, Sydney, New South Wales Australia; 67 School of Public Health, Imperial College London, London, UK; 68 Ibaraki Western Medical Center, Chikusei, Japan; 69 Department of Public Health, Kindai University Faculty of Medicine, Sakai, Japan; 70 Institute for Global Health Policy Research, Japan Institute for Health Security, Tokyo, Japan; 71 Department of Nephrology, Ambroise Paré University Hospital, APHP, Paris, France; 72 Stanford Center for Clinical Research, Department of Medicine, Stanford University, Stanford, CA USA; 73 NHMRC Clinical Trials Centre, University of Sydney, Sydney, New South Wales Australia; 74 Division of Nephrology, Department of Medicine, University of Maryland Medical Center, Baltimore, MD USA; 75 Division of Nephrology, Charles and Jane Pak Center for Mineral Metabolism and Clinical Research, University of Texas Southwestern Medical Center, Dallas, TX USA; 76 Department of Epidemiology, Peter O’Donnell Jr School of Public Health, University of Texas Southwestern Medical Center, Dallas, TX USA; 77 Division of Nephrology, Department of Medicine, College of Medicine, University of Illinois Chicago, Chicago, IL USA; 78 Division of Nephrology, Department of Medicine, University of Michigan, Ann Arbor, MI USA; 79 Division of Cardiology, Department of Internal Medicine, University of Texas Southwestern Medical Center, Dallas, TX USA; 80 Interdisciplinary Data Science, Duke University, Durham, NC USA; 81 Postgraduate Program in Epidemiology, Universidade Federal do Rio Grande do Sul, Porto Alegre, Brazil; 82 Department of Statistics, Universidade Federal do Rio Grande do Sul, Porto Alegre, Brazil; 83 National School of Public Health, Oswaldo Cruz Foundation, Rio de Janeiro, Brazil; 84 Centro de Pesquisa Clínica e Epidemiológica, Hospital Universitário, Universidade São Paulo, São Paulo, Brazil; 85 Instituto Gonçalo Moniz (IGM), Fundação Oswaldo Cruz (Fiocruz), Salvador, Brazil; 86 Faculty of Medicine & Clinical Hospital/EBSERH, Universidade Federal de Minas Gerais, Belo Horizonte, Brazil; 87 Institute of Epidemiology and Prevention, Faculty of Medicine and Medical Center, University of Freiburg, Freiburg, Germany; 88 Nephrology and Primary Care, Department of Medicine IV, Faculty of Medicine and University Medical Center, University of Freiburg, Freiburg, Germany; 89 Department of Medicine IV, Faculty of Medicine and University Medical Center, University of Freiburg, Freiburg, Germany; 90 Berta-Ottenstein Program, Faculty of Medicine, University of Freiburg, Freiburg, Germany; 91 Department of Nephrology and Hypertension, Friedrich-Alexander-Universität Erlangen-Nürnberg, Erlangen, Germany; 92 Department of Nephrology and Clinical Immunology, University Hospital RWTH Aachen, Aachen, Germany; 93 Division of Population Health and Genomics, School of Medicine, Ninewells Hospital & Medical School, University of Dundee, Dundee, UK; 94 School of Medicine, University of St Andrews, St Andrews, UK; 95 Vascular and Renal Translational Research Group, Biomedical Research Institute of Lleida Fundació Dr. Pifarré (IRBLleida), Lleida, Spain; 96 Department of Basic Medical Sciences, University of Lleida, Lleida, Spain; 97 RICORS 2040, Instituto de Salud Carlos III, Madrid, Spain; 98 Department of Experimental Medicine, University of Lleida, Lleida, Spain; 99 Department of Nephrology, Arnau de Vilanova University Hospital, Lleida, Spain; 100 Department of Cardiovascular Sciences, University of Leicester, and John Walls Renal Unit, Leicester General Hospital, University Hospitals of Leicester NHS Trust, Leicester, UK; 101 Herbert Wertheim School of Public Health and Human Longevity Science, University of California, San Diego, La Jolla, CA USA; 102 Division of Nephrology–Hypertension, Department of Medicine, University of California, San Diego, La Jolla, CA USA; 103 Nephrology Section, VA San Diego Healthcare System, San Diego, CA USA; 104 Division of Nephrology, Department of Medicine, VA Greater Los Angeles Healthcare System, Los Angeles, CA USA; 105 Department of Medicine, David Geffen School of Medicine, University of California, Los Angeles, Los Angeles, CA USA; 106 Departments of Medicine and Epidemiology, University of Alabama at Birmingham, Birmingham, AL USA; 107 Division of Nephrology, Boston University School of Medicine, Boston, MA USA; 108 Singapore Eye Research Institute, Singapore National Eye Centre, Singapore, Singapore; 109 Ophthalmology and Visual Sciences Academic Clinical Program (Eye ACP), Duke-NUS Medical School, Singapore, Singapore; 110 Donal O’Donoghue Renal Research Centre, Department of Renal Medicine, Northern Care Alliance NHS Foundation Trust, Salford, UK; 111 Faculty of Biology, Medicine and Health, University of Manchester, Manchester, UK; 112 Julius Center for Health Sciences and Primary Care, University Medical Center Utrecht Utrecht University, Utrecht, The Netherlands; 113 Nephrology, University Medical Centre Utrecht, Utrecht, The Netherlands; 114 Department of Internal Medicine, University of California Davis, Sacramento, CA USA; 115 Department of Internal Medicine, Max Rady College of Medicine, University of Manitoba, Winnipeg, Manitoba Canada; 116 Section of Clinical Chemistry, Department of Medical Sciences, Uppsala University, Uppsala, Sweden; 117 Department of Public Health and Caring Sciences, Molecular Geriatrics, Uppsala University, Uppsala, Sweden

**Keywords:** Epidemiology, Cardiovascular diseases, Chronic kidney disease, Risk factors

## Abstract

The American Heart Association’s PREVENT equations estimate risk of total cardiovascular disease (CVD), atherosclerotic cardiovascular disease (ASCVD) and heart failure (HF) to guide lipid-lowering and blood pressure-lowering therapy in people ages 30−79 years in the United States. The SCORE2 risk algorithm is used to estimate CVD risk for similar purposes in people ages 40 and older in Europe. Neither set of equations has been comprehensively validated in global observational cohorts and randomized trials. In this study, in 44 observational cohorts and 18 randomized trials, we assessed discrimination and calibration of the two risk algorithms across geographical regions (North America, Europe and Asia/Other or multiregional trials). We also created scaling factors for risk prediction over 1–9 years using the PREVENT equations, enabling shorter-term risk prediction for research purposes or to facilitate clinical trial enrollment. Over 5.1 years of mean follow-up, 293,737 PREVENT total CVD events (fatal and non-fatal ASCVD or HF) and 258,086 SCORE2 CVD events (myocardial infarction, stroke or cardiovascular death) were observed among 6,422,714 and 5,437,384 individuals, respectively. Despite differences in CVD outcome definitions, target populations and predictor variables, overall discrimination and calibration were similar for both equations, with generally good performance across regions, including in multiregional randomized trials. These findings lend support for adoption of PREVENT or SCORE2 for cardiovascular risk stratification across diverse settings.

## Abstract (web summary)

Comprehensive, multinational validation of the PREVENT and SCORE2 cardiovascular risk scores, used in the United States and Europe, respectively, in 44 observational studies and 18 randomized trials, shows similar performance for the two risk scores and generally good performance across geographical regions.

## Main

The primary purpose of cardiovascular risk prediction tools is to guide patient counseling by using absolute risk estimates to inform the initiation of preventive treatments. These tools are particularly valuable for primary prevention in individuals without CVD, because the distribution of risk in this population is broad. Accurate identification of those at high absolute CVD risk enables targeted use of preventive therapies, such as lipid-lowering medications and more intensive blood pressure targets, to optimize both risk−benefit and cost-effectiveness^1^. Communicating absolute risk estimates can also be a powerful motivator for patients to adopt non-pharmacological measures, including smoking cessation, physical activity and healthier dietary habits.

Among the many available cardiovascular risk prediction models, the Predicting Risk of Cardiovascular Disease EVENTs (PREVENT) and Systematic Coronary Risk Evaluation 2 (SCORE2) tools are notable for their development and validation in large, contemporary cohorts and for their endorsement by major professional societies in the United States and Europe. PREVENT, published in 2023, was developed in more than three million individuals from North America to estimate 10-year and 30-year risks of total CVD, ASCVD and HF. Likewise, SCORE2 provides 10-year CVD risk estimates based on extensive data from European cohorts and underpins prevention recommendations in European guidelines. Both tools have undergone substantial validation within their regions of origin.

Despite their widespread use, each equation was primarily developed and validated in region-specific populations: PREVENT in North America and SCORE2 in Europe. As a result, neither tool has been robustly evaluated in globally diverse populations with different baseline risk profiles or in large multinational randomized trials that recruit individuals across a broad range of countries and care settings. This limits confidence in their broader applicability and raises uncertainty about their generalizability outside the populations in which they were derived.

Establishing the validity of these equations across heterogeneous clinical and geographical contexts is essential to support wider, more consistent implementation of risk-based cardiovascular prevention. To address this evidence gap, we conducted a comprehensive multinational validation of the PREVENT and SCORE2 equations across observational cohorts spanning a wide range of baseline cardiovascular risk and geographical regions as well as across diverse multinational randomized clinical trials. We evaluated both discrimination and calibration to determine how these models perform in a global context.

## Results

**Fig. 1:**
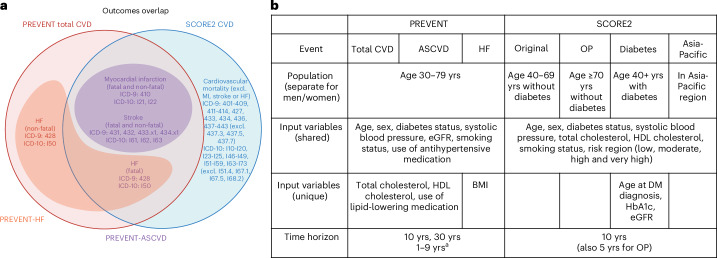
Target population, input variables, time horizon and outcomes for the PREVENT and SCORE2 equations. **a**, Venn diagram of the overlap in outcome definitions for PREVENT and SCORE2 equations. **b**, Review of the populations, variables and time horizon for risk prediction for each of the equations. Venn diagram created in BioRender; Ballew, S. https://biorender.com/u8ie6j7 (2026). ^a^Scaling factors for calculation of risk over shorter time horizons (1−9 years) for the PREVENT equations are detailed in the Supplementary Methods. For example, the 5-year CVD risk from the PREVENT base model equals exp(−0.858) × 10-year risk / (1 − 10-year risk + exp(−0.858) × 10-year risk). 10-year risks of 2.5%, 5%, 10% and 20% convert to 5-year risks of 1.1%, 2.2%, 4.5% and 9.6%, respectively. BMI, body mass index; DM, diabetes mellitus; eGFR, estimated glomerular filtration rate; excl., excluding; HDL, high-density lipoprotein; ICD-9, International Classification of Diseases, Ninth Revision; ICD-10; International Classification of Diseases, Tenth Revision; MI, myocardial infaction; OP, SCORE2 Older Persons; yrs, years.

The overlap and distinction among PREVENT and SCORE CVD populations, predictor variables and outcome definitions are displayed in Fig. 1. Scaling factors used to estimate PREVENT risk over shorter time horizons (1−9 years), with the 5-year PREVENT risk equation provided as an example, are shown in the Supplementary Methods.

For evaluation of PREVENT equations, we included data from 6,422,714 individuals without CVD at baseline across 62 studies: 6,307,652 from 31 North American observational cohorts, 18,749 from eight European observational cohorts, 43,311 from five Asian/Other observational cohorts and 53,002 from 18 multiregional randomized trials (Table 1). Of these, 20 studies (*n* = 4,408,135), all observational cohorts from North America, were included in the original PREVENT validation (but not development), and 42 studies were new (*n* = 2,014,579) (Supplementary Table 1). In the 18 randomized trials, 13,679 (26%) individuals were recruited from North America, 18,161 (34%) were recruited from Europe and 21,162 (40%) were recruited from Asia or other regions. For evaluation of SCORE2, two studies (*n* = 13,688) were original validation studies of SCORE2 development, and 58 studies (*n* = 5,423,696) were new (Supplementary Table 2).

**Table 1:** Baseline characteristics of included cohorts and trials for PREVENT evaluation, stratified by validation status and geographical location

	Overall (62)	Original PREVENT validation studies (20)	New PREVENT validation studies			
			North America (11)	Europe (8)	Asia/Other(5)	Multiregional trials (18)
*n*	6,422,714	4,408,135	1,899,517	18,749	43,311	53,002
Follow-up, years, mean (s.d.)	5.1 (3.3)	5.5 (3.4)	4.2 (2.5)	7.2 (4.5)	9.9 (5.3)	3.4 (1.6)
Age, years, mean (s.d.)	55 (13)	53 (13)	59 (12)	61 (12)	54 (10)	63 (9)
Female, %	46%	57%	21%	42%	52%	39%
Total chol, mmol l^−1^, mean (s.d.)	5.0 (0.9)	5.0 (0.9)	4.9 (0.9)	5.1 (1.1)	5.4 (0.9)	5.0 (1.0)
Non-HDL-c, mmol l^−1^, mean (s.d.)	3.7 (0.9)	3.6 (0.9)	3.7 (0.9)	3.8 (1.1)	4.0 (1.0)	3.7 (1.0)
HDL-c, mmol l^−1^, mean (s.d.)	1.3 (0.4)	1.4 (0.4)	1.2 (0.4)	1.4 (0.4)	1.4 (0.4)	1.2 (0.3)
SBP, mmHg, mean (s.d.)	127 (16)	126 (16)	129 (16)	140 (19)	129 (18)	139 (17)
Diabetes, %	15%	11%	21%	39.3%	12%	75%
Current smoking, %	17%	15%	20%	20%	18%	15%
BMI, kg m^−2^, mean (s.d.)	29 (5)	29 (5)	29 (5)	28 (5)	25 (4)	29 (5)
Antihypertension medication use, %	24%	29%	11%	73%	20%	86%
Statin use, %	18%	14%	26%	46%	8.7%	42%
eGFR, ml min^−1^ 1.73 m^−^^2^, mean (s.d.)	88 (18)	90 (18)	86 (16)	68 (27)	91 (16)	65 (26)
ACR missing, %	62%	54%	84%	39.4%	6.2%	5.6%
UACR, mg g^−1^, median (IQR)	8 (8−12)	8 (8−12)	9 (8−17)	50 (9−357)	7 (5−9)	210 (45−751)
Age at diabetes, years (s.d.)*	59 (12)	58 (12)	59 (10)	57 (13)	NA	51 (11)
HbA1c in DM, %, mean (s.d.)	7.4 (1.9)	7.5 (1.8)	7.3 (1.9)	7.4 (1.4)	7.1 (1.7)	7.9 (1.5)
PREVENT total CVD, *n*	293,737	184,174	103,515	2,164	107	3,777
PREVENT ASCVD, *n*	180,195	116,537	57,418	1,560	1,677	3,003
PREVENT HF, *n*	166,514	105,602	58,758	885	22	1,247

Studies are divided into those used in the original validation of the PREVENT score^9^ and in the current, new validation. Number of cohorts and trials in each risk group/region is displayed in parentheses. There were 57 cohorts included in PREVENT total CVD analyses, 62 in PREVENT ASCVD analyses and 52 in PREVENT HF analyses. *Age at diabetes only within individuals with diabetes.

BMI, body mass index; chol, cholesterol; DM, diabetes mellitus; eGFR, estimated glomerular filtration rate; HDL-c, high-density lipoprotein cholesterol; NA, not available; SBP, systolic blood pressure.

Mean age of participants across studies included in the PREVENT equation evaluation ranged from 53 years to 63 years across geographical regions. Women represented 39% of the sample from trials compared to approximately 46% in observational cohorts. The prevalence of diabetes and chronic kidney disease (CKD) and use of antihypertensive medications and statins were all higher in randomized trials than in observational cohorts. Participant characteristics of individual study populations used in SCORE2 evaluation, ages 40−89, are summarized in Supplementary Table 3.

**Fig. 2:**
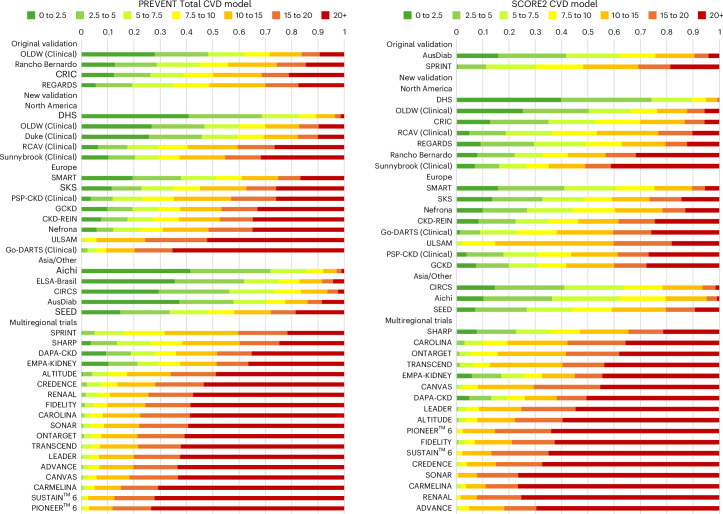
Distribution of 10-year predicted risks for the PREVENT total CVD and SCORE2 CVD equations. Predicted risks are shown by study and stratified by validation status and geographical location.

The distribution of predicted PREVENT total CVD and SCORE2 CVD risk at baseline within studies, stratified by geographical region and presence as an original validation cohort, is shown in Fig. 2. Participants in European cohorts generally had a higher baseline predicted total CVD risk than participants in North American cohorts and Asian/Other cohorts. Participants in clinical trials also had a high predicted CVD risk (Supplementary Tables 1 and 3). The distributions of predicted risk for PREVENT ASCVD and HF events were similar to PREVENT total CVD (Supplementary Fig. 1**)**.

For the PREVENT outcomes, over a mean follow-up of 5.1 years, 293,737 individuals experienced a total CVD event, 180,195 experienced an ASCVD event and 166,514 had an HF event. For the SCORE2 outcome, over a mean follow-up of 5.2 years, 258,086 individuals experienced a SCORE2 CVD event.

**Fig. 3:**
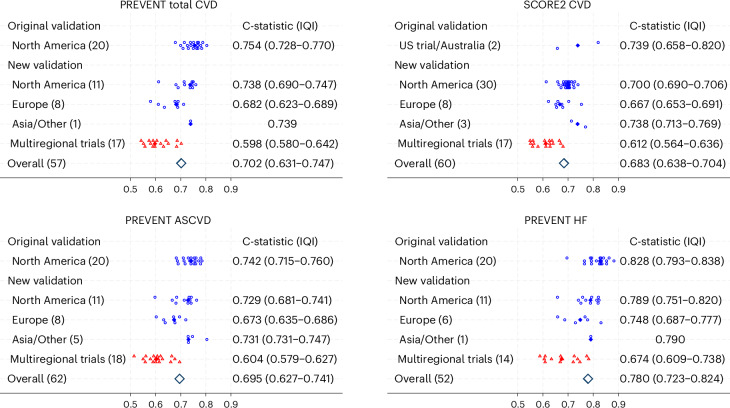
C-statistics for PREVENT total CVD, SCORE2 CVD, PREVENT ASCVD and PREVENT HF. C-statistics are stratified by validation status and geographical location. Median C-statistics are presented with corresponding IQIs. Observational studies are represented as blue circles and trials as red triangles. The number of studies within each category is denoted as the number within the parentheses. Median C-statistics in each risk group and geographical region are displayed as solid diamonds.

Discrimination was moderate to favorable overall for both equations (Fig. 3). Median C-statistics were 0.702 (interquartile interval (IQI): 0.631−0.747) for PREVENT total CVD and 0.683 (IQI: 0.638−0.704) for SCORE2 CVD. For ASCVD and HF events, discrimination was also satisfactory overall (0.695 (IQI: 0.627−0.741) and 0.780 (IQI: 0.723−0.824), respectively). Discrimination performance was generally consistent across geographical regions for both PREVENT and SCORE2 equations (Supplementary Table 4); however, discrimination differed according to the baseline-predicted cardiovascular risk profile of studies. When examined continuously across mean baseline-predicted cardiovascular risk of each study, discrimination was superior for all outcomes in lower-risk studies (all *P*< 0.001); however, declines in the observed Harrellʼs C-statistic closely paralleled declines in the model-based C-statistic (the maximum discrimination expected given the cohort’s case-mix), indicating that differences in discrimination were largely attributable to case-mix heterogeneity (Supplementary Fig. 2). After adjusting for baseline-predicted risk, discrimination performance of the PREVENT equations was similar by sex (*P*> 0.05 for all; Supplementary Fig. 3). Discrimination performance was also consistent across active and control arms in the randomized trials (Supplementary Fig. 4).

**Fig. 4:**
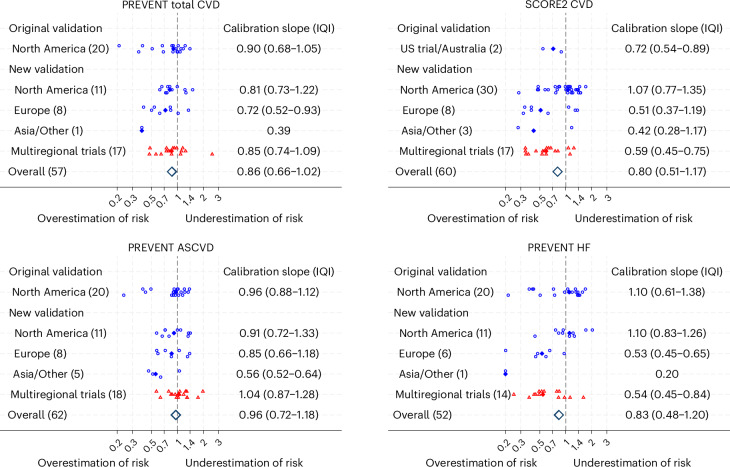
Calibration slopes for PREVENT total CVD, SCORE2 CVD, PREVENT ASCVD and PREVENT HF. Calibration slopes are stratified by validation status and geographical location. Median calibration slopes are presented with corresponding IQIs. Observational cohorts are represented as circles and trials as triangles. Median calibration slopes in each risk group and geographical region are displayed as solid diamonds. Calibration slope was estimated from a regression of observed risk on predicted risk (for example, a slope <1.0 indicates overprediction, where observed risk is lower than predicted risk).

Calibration slopes indicated that PREVENT modestly overestimated risk for total CVD (median calibration slope 0.86 (IQI: 0.66−1.02)) as did SCORE2 (0.80 (IQI: 0.51−1.17)). PREVENT calibration was favorable for ASCVD events (0.96 (IQI: 0.72−1.18)) and HF events (0.83 (IQI: 0.48−1.20)) (Fig. 4). For total CVD and ASCVD, calibration was generally consistent across geographical regions, with the potential exception of Asia/Other regions where there were limited data but a suggestion of overprediction (PREVENT total CVD, one study, slope 0.39; PREVENT ASCVD, five studies, slope 0.56 (0.52−0.64); SCORE2, three studies, 0.42 (0.28−1.17)). For HF, calibration slope was also lower (in other words, observed risk was lower than predicted risk) outside of North America. However, calibration for PREVENT equations was consistent across geographical regions in analyses restricted to multinational randomized trials, where outcomes are generally adjudicated, as well as across active and control arms (Supplementary Fig. 4).

The PREVENT score with albuminuria improved discrimination for total CVD (meta-analyzed change in C-statistic (0.014 (95% confidence interval: 0.012−0.015)), ASCVD 0.009 (0.008−0.010)) and HF (0.004 (0.003−0.005)) compared to the PREVENT base model (all *P* < 0.0001; Table 2). Across all three outcomes, there was an interaction by baseline risk, such that the greatest magnitude of improvement was observed in high-risk studies that predominantly included individuals with diabetes and/or CKD (all *P* < 0.05). Calibration of PREVENT was generally similar between the base and albuminuria models (Supplementary Table 5). Discrimination was slightly improved with the addition of glycated hemoglobin (HbA1c) to the PREVENT base model (Table 2 and Supplementary Table 5), with no interaction by baseline risk status (all *P* > 0.05).

**Table 2:** Meta-analyzed change in C-statistics and 95% confidence interval with the PREVENT-albuminuria or PREVENT-HbA1c equation compared to the PREVENT-base model

Change in C-statistic by equation	PREVENT total CVD	PREVENT ASCVD	PREVENT HF
**PREVENT-albuminuria**	0.014 (0.012−0.015)	0.009 (0.008−0.010)	0.004 (0.003−0.005)
**PREVENT-HbA1c**	0.003 (0.001−0.004)	0.003 (0.002−0.004)	0.002 (0.001−0.002)

C-statistics and change in C-statistics for individual studies are provided in Supplementary Tables 5 and 6. There were 57 cohorts included in PREVENT total CVD analyses, 62 in PREVENT ASCVD analyses and 52 in PREVENT HF analyses.

As a sensitivity analysis, we compared the performance of the PREVENT ASCVD equation with the pooled cohort equations (PCE). Overall, C-statistics were broadly similar; studies with lower discrimination for PREVENT also demonstrated lower discrimination for PCE (Supplementary Fig. 5). However, calibration was consistently inferior for PCE compared to PREVENT, with systematically lower calibration slopes, meaning the observed risk was lower than predicted risk (Supplementary Fig. 5). Calibration slopes for PREVENT, SCORE2 and PCE by individual cohort and trial are shown in Supplementary Fig. 6.

## Discussion

In what is, to our knowledge, the largest global assessment of the PREVENT and SCORE2 cardiovascular risk equations to date, we made several observations with notable implications for both clinical implementation and future model refinement. Overall, discrimination for both equations was favorable across a broad range of populations and settings, including clinical trials. Although performance was generally consistent across geographical regions, discrimination was stronger in low-risk and intermediate-risk populations—findings that support prioritizing the use of these tools in primary care, where risk-based decisions are most relevant. We also observed modest overprediction for certain outcomes and in specific geographical regions or cohorts, suggesting that ongoing refinement, including region-specific adaptations, may further enhance calibration. Finally, incorporating albuminuria into PREVENT improved discrimination, underscoring the clinical value of this underutilized biomarker. Together, these findings support broader global implementation of both PREVENT and SCORE2, especially in primary care, while also highlighting opportunities to further strengthen risk stratification by integrating albuminuria where available.

A persistent barrier to the widespread adoption of risk prediction tools is uncertainty around their generalizability across diverse clinical and population settings^2^. Our finding that both PREVENT and SCORE2 demonstrated favorable discrimination across multiple geographical regions and clinical trials suggests that each equation may have broader applicability than originally envisioned. The superior calibration of PREVENT over the PCE supports its adoption as the preferred CVD risk prediction tool in the United States. More broadly, by combining multiple outcomes within a single tool, PREVENT simplifies implementation for clinicians and aligns with the growing need for a holistic approach to cardiovascular−kidney−metabolic multimorbidity, particularly HF prevention, where newer therapies offer considerable potential to alter disease trajectory. We also present coefficients that allow prediction for 1−9 years of risk. The multiple iterations of SCORE2, such as SCORE2-OP for older adults^3^, SCORE2-Diabetes for individuals with diabetes^4^ and SCORE2 Asia-Pacific^5^, support a more harmonized global implementation of risk-based cardiovascular prevention under one family of risk equations. This analysis represents an important step toward more consistent and scalable deployment of these tools worldwide.

The observation that discrimination was higher in lower-risk cohorts has implications for implementation. In community-based settings, individuals exhibit a wide range of cardiovascular risk profiles, allowing both PREVENT and SCORE2 to more effectively differentiate higher-risk from lower-risk individuals. By contrast, populations enriched for people at high baseline risk (for example, cardiovascular outcome trials) show a much narrower distribution of predicted risk (on the multiplicative or log scale), which inherently limits the achievable C-statistic for a given model structure. We demonstrated this analytically by showing that the constrained maximum possible discrimination based on the degree of risk factor heterogeneity within a cohort explains much of the variation in observed discrimination across cohorts. These findings reinforce that both PREVENT and SCORE2 are most informative in primary care and other lower-risk settings, where population heterogeneity enhances model discrimination and where risk-based decisions are most clinically impactful. The equations are well calibrated for higher-risk groups and trials, allowing for coherent conclusions across the full range of risk as well as screening for clinical trial eligibility globally.

There was some evidence that PREVENT may overpredict HF risk outside of North America and total CVD and ASCVD risk in Asian/Other regions. The reason for this is not entirely clear. Notably, many of the North American cohorts were drawn from electronic health records (EHRs), whereas this type of cohort was rare among the European and Asian/Other studies. This may have translated to differences in outcome ascertainment or administrative coding practices, particularly for HF with preserved ejection fraction, which is a complex diagnosis often made in outpatients. Alternatively, it may be true that North Americans face a higher burden of obesity with greater risk of obesity-related HF with preserved ejection fraction^6^. However, the more consistent calibration for HF across regions in the trials, where events were typically adjudicated by blinded expert committees, suggests that the former is more likely. Further investigation, particularly in Asia, is warranted to better understand and address this observation, such as local calibration. In the United States, PREVENT includes an optional input for zip code to account for place-based differences in risk due to neighborhood-level deprivation. Similar adaptions outside of North America, as has been done with SCORE2 Asia-Pacific, may further enhance performance.

Validation of both PREVENT and SCORE2 within large multinational randomized trials of contemporary cardiometabolic therapies—including sodium-glucose cotransporter-2 (SGLT2) inhibitors, glucagon-like peptide-1 (GLP-1) receptor agonists and non-steroidal mineralocorticoid receptor antagonists (MRAs)—is particularly valuable for establishing future thresholds for initiating these therapies. These agents have all demonstrated broad benefits across multiple cardiovascular−kidney−metabolic complications, yet clear risk-based initiation thresholds are still evolving. The 2025 United States hypertension guidelines now recommend PREVENT to identify individuals for whom pharmacological blood pressure lowering is recommended: treatment is advised for patients with blood pressure ≥130/80 mmHg and a 10-year PREVENT-estimated CVD risk ≥7.5%. SCORE2 is similarly used within contemporary European guidelines, which tie its risk categories to progressively more intensive recommendations for blood pressure-lowering and lipid-lowering therapies. Developing consensus thresholds for initiating newer cardiometabolic therapies will require further analyses of clinical trial data to generate reliable evidence on the absolute benefits and harms of these therapies across varying levels of predicted risk, assessment of the distribution of PREVENT risk estimates in the United States and other populations and consideration of the population-level implications of any proposed threshold for treatment^7^. Our analysis, which includes major trials across all three drug classes, provides a strong foundation for these efforts.

Key strengths of this work include the breadth and diversity of studies, regardless of kidney function, encompassing general population cohorts, CKD-specific cohorts, multinational randomized controlled trials and EHR-based cohorts. The analysis draws on substantially more studies than were included in the original validation of the PREVENT and SCORE2 equations and represents, to our knowledge, the largest external validation of both equations to date. We also created scaling factors for the PREVENT equations for risk prediction shorter than 10 years, including 5-year risk.

Some limitations should be considered when interpreting these findings. There were differences in average predicted risk of the cohorts by region: the included European cohorts were generally much higher risk than the North American cohorts, which limited our ability to explore geographical variation in tool performance across the full spectrum of baseline-predicted cardiovascular risk. Representation from geographical regions outside of North America and Europe was relatively limited, with little data available for much of the Middle East and Africa. Patterns of CVD may differ across regions; for example, the incidence of stroke, particularly hemorrhagic stroke, in some East Asian countries is higher than in other parts of the world^8^. Thus, further validation of both equations in additional cohorts from Asia and other underrepresented populations is warranted. Nevertheless, the inclusion of 18 international trials, which enrolled approximately 50,000 participants from geographically diverse regions (the majority of whom were recruited outside of North America), partially mitigates this limitation. Finally, individuals may have undertaken treatments or changed behaviors over the course of follow-up; this was not captured in the analytic plan.

In summary, these findings support broader adoption of the PREVENT and SCORE2 equations to facilitate risk-based stratification of individuals for cardiovascular care while also highlighting the value of incorporating albuminuria for more precise risk stratification.

## Methods

### Data sources

This analysis was conducted within the CKD Prognosis Consortium, a collaborative network comprising over 100 studies worldwide, with representation from North and South America, Europe and Asia-Pacific^10^. These include observational cohorts based on EHRs, prospective cohorts drawn from general, high-risk and CKD populations as well as clinical trials of glucose-lowering, blood pressure-lowering and lipid-lowering therapies. The CKD Prognosis Consortium incorporates studies without restrictions based on kidney function, thereby enabling representation of both the general population and individuals with CKD. The institutional review board at New York University Grossman School of Medicine waived the need for informed consent for this secondary data analysis and approved this study.

In this analysis, we included only cohorts and trials that were not part of the original derivation sample for the PREVENT and SCORE2 equations. We reused cohorts previously employed to validate either equation (‘original validation’) and added cohorts and trials not included in previous validation efforts (‘new validation’). As the equations were developed and validated using different datasets, the cohorts classified as original validation or new validation differed by equation. The multinational, randomized, double-blind, placebo-controlled trials had not been used in validation of either equation and evaluated four contemporary cardiovascular−kidney−metabolic therapies: renin-angiotensin system blockade, SGLT2 inhibitors, GLP-1 receptor agonists and non-steroidal MRAs. The trials enrolled participants from approximately 50 countries across North and South America, Europe, the Middle East, Africa and Asia-Pacific. Trials that included patients free of CVD were invited to participate individually or were included if data were publicly available. Identification and coordination of SGLT2 inhibitor trials was facilitated by the SGLT2 Inhibitor Meta-Analysis Cardio-Renal Trialists Consortium^11^, and trials of other drug classes were identified through previously published systematic reviews and meta-analyses^12–16^. Further details on individual studies are provided in the Supplementary Methods.

### PREVENT and SCORE2 risk equation

The PREVENT risk equation estimates 10-year and 30-year risk of total CVD (fatal and non-fatal ASCVD (myocardial infarction or stroke) or HF) in people aged 30−79 years without established CVD. The model incorporates routinely collected demographic, clinical and laboratory data and also estimates absolute risk of ASCVD and HF separately. PREVENT was developed using data from 3,281,919 individuals across 25 North American cohorts. PREVENT also includes optional predictors of kidney and metabolic risk, including albuminuria (as measured by urinary albumin:creatinine ratio (UACR)) and HbA1c, where available. Similar equations are available for ASCVD and HF. Full details of the development and validation of PREVENT were previously published^9^. The equations to predict shorter-term risk of CVD are newly presented here and are available in the Supplementary Methods.

SCORE2 estimates 10-year risk of incident CVD in a primary prevention setting for adults without diabetes between the ages of 40 years and 69 years, with shorter-term risk estimated assuming a constant hazard (Supplementary Methods). A key difference between SCORE2 and PREVENT is the definition of the CVD outcome, which, in SCORE2, is defined as myocardial infarction, stroke or cardiovascular death. Thus, although HF events are not included, deaths due to cardiovascular causes other than myocardial infarction, stroke or HF (for example, arrythmia, sudden cardiac death and other cardiovascular deaths) are incorporated in the main outcome for SCORE2 but not for PREVENT. SCORE2 was derived from a dataset comprising 677,684 individuals enrolled in 45 prospective European cohorts^17^. The model includes population-specific calibrations and extensions, including adaptations for older adults over the age of 70 (SCORE2-OP)^3^, individuals with diabetes (SCORE2-Diabetes)^4^ and those from the Asia-Pacific region, including Australia (SCORE2 Asia-Pacific)^5^.

### Outcomes and study stratification

We evaluated the performance of each model according to how outcomes were defined in model development. Specifically, for PREVENT, we evaluated three outcomes: (1) incident total CVD, defined as a composite of fatal and non-fatal ASCVD events and HF events; (2) ASCVD, comprising fatal and non-fatal myocardial infarction and stroke; and (3) HF events. PREVENT accounts for non-cardiovascular death as a competing risk. For SCORE2, the predicted outcome was incident CVD, defined as a composite of cardiovascular death, non-fatal myocardial infarction and non-fatal stroke. Differences in the definition of incident CVD between the two equations are displayed in Fig. 1.

Because our primary aim was to evaluate model performance across diverse settings, all analyses were stratified by geographical region (North America, Europe and Asia/Other regions or multiregional trials).

### Statistical analysis

Consistent with previous work undertaken by the CKD Prognosis Consortium, we used a harmonized analytical approach applied individually to studies, followed by meta-analysis^18^. Characteristics of participants in each cohort and trial were summarized with mean and standard deviation (or median and IQI if skewed) for continuous variables and proportions for categorical variables.

We evaluated the distribution of predicted risk for the three PREVENT outcomes and SCORE2 incident CVD outcome in each cohort. Where applicable, we applied the relevant SCORE2 model to the target population at the individual participant level. For example, for those with diabetes, the SCORE2-Diabetes model was applied; for those over the age of 70 years without diabetes, the SCORE2-OP equation was used; and for those in the Asia-Pacific region, the SCORE2 Asia-Pacific recalibration combined with the appropriate SCORE2 equation was used.

We assessed discrimination and calibration within each study, summarizing as median/interquartile overall and by geographical region. Discrimination was estimated by calculating Harrellʼs C-statistics, up to 10 years, within individual studies, using a standard survival analysis framework (censoring at competing events, such as loss to follow-up or death from causes not in the specific outcome of interest). Calibration was assessed using calibration slopes within each study, using competing risk methodology. Observed risks were generated using pseudo-observations of the cumulative incidence up to 10 years, accounting for censoring and the competing event of non-cardiovascular death, and then slopes were estimated using generalized linear models to regress the pseudo-observations on predicted risks, assuming a zero intercept. For discrimination and calibration assessment, the timeframe for predicted risk was tailored to each study, determined using the length of follow-up at the 95th percentile for events, with a maximum of 10 years. Predicted PREVENT risks for 1−9 years were estimated using scaling factors of the 10-year risk (details in Supplementary Methods), and, for SCORE2, shorter-term risk was estimated assuming a constant hazard (Supplementary Methods).

In additional analyses, we evaluated discrimination according to mean predicted CVD risk at the study level. We examined the extent to which case-mix heterogeneity constrained the maximum achievable discrimination at higher predicted risk by plotting model-based (case-mix-determined maximum achievable) discrimination versus mean predicted risk and the observed C-statistic versus model-based discrimination. To evaluate whether differences in discrimination by geographical region persisted after accounting for mean baseline-predicted cardiovascular risk, we performed meta-regression of the C-statistic on the mean baseline-predicted cardiovascular risk as well as geographical region (North America, Europe and Asia/Other and multiregional trials). We repeated these analyses by sex.

In additional analyses, we evaluated the improvement in C-statistics with the addition of albuminuria and HbA1c to each PREVENT outcome. For individuals for whom only urinary protein:creatinine ratio (PCR) was available, these values were converted to albumin:creatinine ratio (ACR) using the unadjusted equation previously developed and validated by the CKD Prognosis Consortium^19^. For individuals for whom only urine dipstick was available, a similar approach was used. Although we previously developed modifications to SCORE2 and SCORE2-OP to allow the addition of kidney function and albuminuria^20^, these modifications have not been developed for the offshoots of SCORE2 (that is, SCORE2-Diabetes and SCORE2 Asia-Pacific), and, thus, it was not possible to incorporate albuminuria for all participants for whom SCORE2 was applied. Therefore, we limited these analyses to PREVENT. Changes in C-statistic were estimated within studies and then meta-analyzed using random effects models. We evaluated for interaction by mean baseline-predicted study risk using meta-regression across studies.

In a sensitivity analysis, we evaluated the discrimination and calibration of the PCE, the current reference standard in the United States, as described above^21^. We related the C-statistics and calibration slope estimated using the PREVENT ASCVD equation and the PCE using Pearsonʼs correlation. All statistical analyses were performed in Stata 18.0.

### Reporting summary

Further information on research design is available in the Nature Portfolio Reporting Summary linked to this article.

## Online content

Any methods, additional references, Nature Portfolio reporting summaries, source data, extended data, supplementary information, acknowledgements, peer review information; details of author contributions and competing interests; and statements of data and code availability are available at 10.1038/s41591-026-04437-z.

## Supplementary information

Supplementary Information Supplementary Methods and listing of supplementary tables and figures in the Excel file

Reporting Summary

Supplementary Tables Excel file with seven supplementary tables and six supplementary figures

## Data Availability

Under agreement with the participating studies, the CKD Prognosis Consortium cannot share individual data with third parties. Inquiries regarding specific analyses should be made to ckdpc@nyulangone.org and will be responded to within 30 days. Investigators may approach the original studies regarding their own policies for data sharing (for example, https://aric.cscc.unc.edu/aric9/researchers/Obtain_Submit_Data for the Atherosclerosis Risk in Communities Study).
