# Supplementary material for: Multinational validation of the PREVENT and SCORE2 cardiovascular risk equations across 6.4 million individuals
Source: Nat Med. 2026 May 5;32(7):2629–38. doi: 10.1038/s41591-026-04437-z (PMC13375529; doi:10.1038/s41591-026-04437-z)
Supplement: Supplementary file 1 [file 41591_2026_4437_MOESM1_ESM.pdf]

nature medicine

Supplementary information

https://doi.org/10.1038/s41591-026-04437-z

Multinational validation of the PREVENT and SCORE2 cardiovascular risk equations across 6.4 million individuals

In the format provided by the authors and unedited

Supplementary Information

Table of Contents

Supplemental Methods. Data analysis overview and 1-9 year PREVENT risk scaling factors, analytic notes for individual cohorts, and further details on covariate and outcome definitions and ascertainment....2

1.1 Overview 2

1.2 Details of the analysis....3

1.3 Notes for individual cohorts 4

1.4 Covariate assessment....6

1.5 Outcome definitions and ascertainment....7

1.5.1 ICD codes used to define outcome if not specified 7

1.5.2 Details of individual studies outcome definitions and ascertainment....7

Listing of Tables and Figures in Excel 12

References 13

Supplemental Methods. Data analysis overview and 1-9 year PREVENT risk scaling factors, analytic notes for individual cohorts, and further details on covariate and outcome definitions and ascertainment

1.1 Overview:

In order to determine the cohorts eligible for this paper, in August 2024 all eligible cohorts in CKD-PC with cardiovascular outcome data available were contacted regarding their interest in this topic and to confirm availability of data. 72 cohorts were contacted, two were not able to participate logistic issues with data sharing and 23 were already included in the original PREVENT derivation, resulting in 47 cohorts from the initial contact. In November/December 2024, we also contacted 20 further cohorts, especially focusing on clinical trial cohorts. One did not have history of cardiovascular disease, and one was not able to run our code within the timeframe to be included in the analyses, adding 18 more cohorts. Upon revision, we removed 3 cohorts that were in the original SCORE2 derivation. There were a total of 62 cohorts included in this analysis.

As previously described,[1] the collaborating cohorts were asked to compile a dataset with approximately 25 variables (Required: main exposure [age, sex, race/ethnicity, history of CVD, smoking, diabetes, systolic blood pressure, antihypertensive medications, total cholesterol, HDL cholesterol, statin use, BMI, eGFR], outcome [fatal and non-fatal myocardial infarction, fatal and non-fatal ischemic and hemorrhagic stroke, fatal and non-fatal heart failure hospitalization, all-cause mortality]; In addition data were captured if available on: albuminuria, and hemoglobin A1c). To be consistent across cohorts, the CKD-PC Data Coordinating Center sent definitions for those variables to participating cohorts. We instructed studies not to impute any variables.

For the 62 cohorts in this specific study, the DCC at NYU Langone Health conducted the final meta-analysis. Then the DCC meta-analyzed the estimates across cohorts using Stata. The CKD-PC data request and processing procedures are as follows. After obtaining opt-in preferences from cohorts for the topics for each phase, the Data Coordinating Center (DCC) requests de-identified data using a specific data request document describing the variables and preferred definitions needed for the current phase of the CKD-PC. Cohorts work with the DCC on any data use agreements, IRB approvals, and other logistic issues for de-identified data transfer. The DCC also advises on any differences in definitions or questions on data formatting. Cohorts then provide de-identified or limited data (in whatever program format, e.g., Stata, SAS, csv) via a secure data transfer provided or their own secure transfer program/platform. Data is stored on a secure password protected network server that is only accessed by limited faculty and staff (<10). All those faculty and staff have completed HIPAA and CITI certification and have signed internal data use agreements to not use the data for any other than stated purposed and to not remove the data from that network drive. The CKD-PC does not share data with any external parties. Once data is received and stored in the network drive, the DCC programmer reviews the data and the data dictionary provided by the cohort to check for any missing information, outliers, and potentials issues with variable units, dates, etc. Any questions are sent to the cohort representatives for data checking and cleaning. Further data checking is done throughout the analysis process for each CKD-PC paper, including a review from a cohort representative of all tables and figures to confirm their cohort representation.

As detailed in our previous reports,[2] each cohort was instructed to standardize their serum creatinine and report its method when available. The reported creatinine standardization allows grouping studies into studies that reported using a standard IDMS traceable method or conducted some serum creatinine standardization to IDMS traceable methods (AusDiab, CAROLINA, CKD-REIN, CREDENCE, CRIC, DAPA-CKD, DHS, ELSA-Brasil, EMPA-KIDNEY, FIDELITY, GCKD, Go-DARTS, LEADER, Rancho Bernardo, RCAV, REGARDS, SEED, SKS, SONAR, SPRINT, SUSTAIN™ 6, TRANSCEND) and studies where the creatinine standardization was not done (ADVANCE, Aichi, CARMELINA, CIRCS, Nefrona, PSP-CKD, RENAAL, SMART, Sunnybrook, ULSAM). For those cohorts without standardization, the creatinine levels were reduced by 5%, the calibration factor used to adjust non-standardized MDRD Study samples to IDMS.[3,4]. We did not adjust creatinine levels in those studies with unknown standardization status (ALTITUDE, CANVAS, Duke, OLDW all cohorts, ONTARGET, PIONEER™ 6, SHARP).

eGFR calculated using the 2021 CKD-EPI creatinine[5]

Sex	Serum Creatinine (mg/dL)	Equation
**CKD-EPI creatinine equation**		
Female	≤0.7	GFR= 142 x $(Scr/0.7)^{-0.241}$ x 0.9938^{Age}
	>0.7	GFR= 142 x $(Scr/0.7)^{-1.200}$ x 0.9938^{Age}
Male	≤0.9	GFR= 142 x $(Scr/0.9)^{-0.302}$ x 0.9938^{Age}
	>0.9	GFR= 142 x $(Scr/0.9)^{-1.200}$ x 0.9938^{Age}

The selection of knots for eGFR, systolic blood pressure and body mass index was based on clinical thresholds.[6]

1.2 Details of the analysis:

Discrimination was calculated using time-dependent Harrel's C-statistic. Calibration slope was calculated by Pseudo-observed risk vs. predicted risk with a slope of 1.0 indicating good calibration (<1 indicating over-prediction and >1 indicating under-prediction). Improvement in C-statistic between the PREVENT base model and ACR or HbA1c models were tested based on a Wilcoxon matched pair rank-sum test of the paired comparisons.

Calibration slopes applied minimum criteria to focus on reliable data. Specifically, the follow-up period was shortened from 10 years to the 95th percentile for events if it was less than 10 years (rounded as 1-9 years). The scaling factors detailed below were used in this instance.

1.2.1 Scaling factors for calculating PREVENT 1-9 years risk from the simplified PREVENT 10-year model*

Follow up, yrs	Base Model					ACR Model					HbA1c Model					Full Model				
	CVD	ASCVD	HF	MI	Stroke	CVD	ASCVD	HF	MI	Stroke	CVD	ASCVD	HF	MI	Stroke	CVD	ASCVD	HF	MI	Stroke
1	-2.670	-2.622	-2.719	-2.633	-2.608	-2.672	-2.623	-2.726	-2.635	-2.611	-2.666	-2.618	-2.721	-2.626	-2.606	-2.663	-2.615	-2.714	-2.627	-2.604
2	-1.937	-1.894	-1.981	-1.903	-1.881	-1.939	-1.895	-1.987	-1.905	-1.884	-1.933	-1.890	-1.982	-1.897	-1.879	-1.930	-1.887	-1.976	-1.898	-1.877
3	-1.491	-1.453	-1.530	-1.461	-1.442	-1.493	-1.454	-1.535	-1.463	-1.444	-1.488	-1.449	-1.531	-1.456	-1.440	-1.485	-1.447	-1.526	-1.456	-1.438
4	-1.163	-1.130	-1.196	-1.137	-1.120	-1.164	-1.130	-1.201	-1.138	-1.122	-1.160	-1.126	-1.197	-1.132	-1.118	-1.158	-1.125	-1.193	-1.132	-1.117
5	-0.858	-0.871	-0.927	-0.877	-0.863	-0.900	-0.872	-0.931	-0.878	-0.864	-0.896	-0.868	-0.928	-0.873	-0.861	-0.894	-0.867	-0.924	-0.873	-0.860
6	-0.676	-0.653	-0.698	-0.658	-0.646	-0.677	-0.653	-0.701	-0.659	-0.647	-0.674	-0.651	-0.699	-0.654	-0.645	-0.672	-0.649	-0.696	-0.655	-0.644
7	-0.480	-0.463	-0.497	-0.467	-0.458	-0.481	-0.464	-0.500	-0.467	-0.459	-0.479	-0.461	-0.498	-0.464	-0.457	-0.478	-0.460	-0.495	-0.464	-0.456
8	-0.306	-0.294	-0.317	-0.296	-0.291	-0.306	-0.294	-0.319	-0.297	-0.291	-0.305	-0.293	-0.317	-0.295	-0.290	-0.304	-0.292	-0.316	-0.295	-0.289
9	-0.147	-0.141	-0.152	-0.142	-0.139	-0.147	-0.141	-0.153	-0.117	-0.139	-0.146	-0.140	-0.153	-0.141	-0.139	-0.146	-0.140	-0.152	-0.141	-0.138

*y-year risk = exp(scaling factor) * 10-year risk /(1 - 10-year risk + exp(scaling factor) * 10-year risk)

Scaling factors per year y (y=1-9) are estimated as the intercept from a regression of logit(PREVENT y-year risk) on logit(PREVENT 10-year risk). The regressions assume a slope of 1.0. PREVENT y-year risk came from the cause-specific model (Khan et.al. Circulation 2023; Table 2) and the PREVENT 10-year risk came from the simplified model (Khan et.al. Circulation 2023; Supplemental Table S24).

CVD: cardiovascular disease; ASCVD: atherosclerotic cardiovascular disease; HF: heart failure; MI: myocardial infarction; ACR: urine albumin to creatinine ratio

Example illustrating calculations for y = 5 for the PREVENT base model for CVD:

PREVENT base model CVD 5-year risk = exp(-0.858) * 10-year risk /(1 - 10-year risk + exp(-0.858) * 10-year risk)

A 10-year risk of 2.5%, 5%, 10%, and 20% converts to 1.1%, 2.2%, 4.5%, and 9.6% respectively. Thus, 5-year risk is slightly less than half of the 10-year risk.

1.2.2 Short-term risk for SCORE2 equations
We estimated using an exponential function of base survival (assuming a constant hazard): y-year base survival = 10-year base survival ^(y/10).

1.3 Notes for individual cohorts:

Cohort	Region	Study Design	Analysis Baseline Year(s)	Additional notes
Aichi	Asia/Other	Research cohort	2002-03	
AusDiab	Asia/Other	Research cohort	1999-2012	
CIRCS	Asia/Other	Research cohort	1986-2010	
CKD-REIN	Europe	Research cohort	2013-16	
CRIC	North America	Research cohort	2003-07	Visit 1 was used as the baseline.
DHS	North America	Research cohort	2000-02	Visit 1 was used as the baseline
Duke	North America	Healthcare administrative database	2016-17	
ELSA-Brasil	Asia/Other	Research cohort	2008-10	
GCKD	Europe	Research cohort	2010-18	
Go-DARTS	Europe	Clinical research cohort	2010-22	
Nefrona	Europe	Research cohort	2009-12	
OLDW	North America	Healthcare administrative database	2010-24	This study used de-identified electronic health record (EHR) data from the Optum Labs Data Warehouse (OLDW). The database contains longitudinal health information on enrollees and patients, representing a mixture of ages and geographical regions across the United States. The EHR-derived data includes a subset of EHR data that has been normalized and standardized into a single database.[7] Cohort inclusion criteria was more than 50 events of any outcome before excluding missing values of main exposure variables. Smoking status might be under measured in this study. All outcomes were defined by ICD codes from encounters. No cause of death information available. Cardiovascular mortality was defined by any hospitalization with related ICD codes and died within 30 days. Baseline index date was set as the earliest date of a serum creatinine measurement after 2008 and at least one year after enrollment.
PSP-CKD	Europe	Clinical database	2006-13	
Rancho Bernardo	North America	Research cohort	1992-97	Visit 1 was used as the baseline.

Cohort	Region	Study Design	Analysis Baseline Year(s)	Additional notes
RCAV	North America	Healthcare administrative database	2004-11	No cause of death information available. Cardiovascular mortality was defined by any hospitalization with related ICD codes and died within 30 days. Baseline index date was set as the earliest date of a serum creatinine at least one year after enrollment.
REGARDS	North America	Research cohort	2003-07	Visit 1 was used as the baseline.
SEED	Asia/Other	Research cohort	2004-11	
SKS	Europe	Research cohort	2002-16	
SMART	Europe	Research cohort	1996-2018	
Sunnybrook	North America	Clinical database	2001-08	
ULSAM	Europe	Research cohort	1991-95	
**Trials**				
ADVANCE	Multi	Clinical trial	2001-03	
ALTITUDE	Multi	Clinical trial	2013	
CANVAS	Multi	Clinical trial	2016-17	
CARMELINA	Multi	Clinical trial	2013-18	History of stroke was not available.
CAROLINA	Multi	Clinical trial	2010-13	
CREDENCE	Multi	Clinical trial	2014	
DAPA-CKD	Multi	Clinical trial	2017	
EMPA-KIDNEY	Multi	Clinical trial	2019-21	
FIDELITY	Multi	Clinical trial	2015-19	Combined dataset of the Phase III studies FIDELIO-DKD and FIGARO-DKD.
LEADER	Multi	Clinical trial	2010-12	
ONTARGET	Multi	Clinical trial	2001-05	History of heart failure was not available. Systolic blood pressure measured at baseline was used.
PIONEER™ 6	Multi	Clinical trial	2017	
RENAAL	Multi	Clinical trial	1996-98	
SHARP	Multi	Clinical trial	2003-06	
SONAR	Multi	Clinical trial	2016	
SPRINT	Multi	Clinical trial	2010-13	
SUSTAIN™ 6	Multi	Clinical trial	2013-14	

Cohort	Region	Study Design	Analysis Baseline Year(s)	Additional notes
TRANSCEND	Multi	Clinical trial	2001-04	History of heart failure was not available. Systolic blood pressure measured at baseline was used.

1.4 Covariate assessment

To be consistent across cohorts, the CKD-PC Data Coordinating Center sent definitions for those medical history variables to participating cohorts (outlined below). We instructed studies not to impute any variables and let us know of any differences in definitions.

**Medical history variable**	**Definition**
Total cholesterol	Total cholesterol (mmol/L)
HDL-Cholesterol	HDL cholesterol (mmol/L)
Systolic blood pressure	Systolic blood pressure (mmHg)
Diabetes mellitus	Glycated hemoglobin A1c ≥6.5% or fasting glucose ≥7.0 mmol/L (≥126 mg/dL) or non-fasting glucose ≥11.1 mmol/L (≥200 mg/dL) or use of glucose lowering drugs (ADA 2010 criteria). Self-report of physician diagnosed diabetes can be included.<br>Identification at any time during study period (from baseline to follow-up).
Hemoglobin A1c (HbA1c)	HbA1C (glycated hemoglobin) (%) measured by a method certified by the National Glycohaemoglobin Standardisation Program (NGSP) and standardized to the Diabetes Control and Complications Trial (DCCT)
Smoking	Information on current-, former-, and never-smoker
Height	Height (m)
Body weight	Body weight (kg)
Medications for hypertension	Taking any medications prescribed for lowering blood pressure. In administrative data we used ATC codes: C02, C03, C07, C08, C09
Statin use	Statin use; In administrative data ATC code C10AA, C10B
Urine albumin-to-creatinine ratio (ACR)	Urinary albumin (mg) / Urinary creatinine (g)
Urine protein-to-creatinine ratio (PCR)	Urinary protein (mg) / Urinary creatinine (g)
Dipstick proteinuria	Dipstick proteinuria
Serum creatinine	Serum creatinine standardized to isotope dilution mass spectrometry (IDMS) (mg/dL) -- specify assay and standardization method

1.5 Outcome definitions and ascertainment

1.5.1 ICD codes used to define outcome if not specified

**Outcome**	**ICD-9 codes**	**ICD-10 codes**	**PREVENT Total CVD**	**SCORE2 CVD**
Myocardial infarction (MI)	410	I21, I22	Y (fatal and non-fatal)	Y (fatal and non-fatal)
Hemorrhagic and Ischemic stroke	431, 432, 433.x1, 434.x1	I61, I62, I63	Y (fatal and non-fatal)	Y (fatal and non-fatal)
Heart failure	428	I50	Y (fatal and non-fatal)	N (only if fatal)
Cardiovascular mortality	401-414, 427-443 (excluding 429.0, 430, 432.1, 435, 437.3, 437.5, 437.7)	I10-I25, I46-I73 (excluding I51.4, I60, I62.0, I67.1, I67.5, I68.2)	N (except if above)	Y

1.5.2 Details of individual studies outcome definitions and ascertainment

**Cohorts**	**Atherosclerotic cardiovascular disease**	**Heart Failure**	**Cardiovascular mortality**	**All-cause mortality**
Aichi	• CHD: fatal MI and non-fatal MI by medical chart review<br>• Stroke: fatal, ischemic and hemorrhagic stroke by medical chart review	NA	Only fatal MI and stroke	
AusDiab	• CHD: MI by ICD codes from hospital discharge<br>• Stroke: ischemic and hemorrhagic stroke by ICD codes from hospital discharge	NA	Cardiovascular mortality by ICD codes from death registry	
CIRCS	• Fatal CHD and MI by medical chart review<br>• Stroke: ischemic and hemorrhagic stroke by a physician panel reviewing medical charts and CT/MRI images for most cases<br>• Fatal stroke by death within 30 days from incident a stroke	NA	Death due to CVD	
CKD-REIN	First episode ever	First episode ever		Data collection based on national registry
CRIC	• CHD: fatal MI and MI adjudicated by a physician panel	Heart failure events were adjudicated by 2 independent		Active and linkage to a registry

Cohorts	Atherosclerotic cardiovascular disease	Heart Failure	Cardiovascular mortality	All-cause mortality
	• Stroke: fatal, ischemic and hemorrhagic stroke adjudicated by a physician panel	physicians who reviewed hospitalization records, and any differences were resolved by discussion.		
DHS	Cardiovascular death, MI, or stroke, adjudicated by cardiologists	Hospitalized heart failure, adjudicated by cardiologists	Death due to CVD	Active and linkage
Duke	• CHD: MI by ICD codes from hospital discharge<br>• Stroke: ischemic and hemorrhagic stroke by ICD codes from hospital discharge	ICD codes from hospital discharge	NA	
ELSA-Brasil	• CHD: fatal MI and non-fatal MI by medical chart review<br>• Stroke: fatal, ischemic and hemorrhagic stroke by medical chart review	Revision of hospitalization records by two independent doctors	Death due to CVD	Active data collection and national mortality registry
GCKD	• Fatal CHD, MI, fatal, ischemic and hemorrhagic stroke, as well as death due to CHD and stroke by medical chart review			
Go-DARTS	• CHD: MI by ICD codes from hospital discharge<br>• Stroke: ischemic and hemorrhagic stroke by ICD codes from hospital discharge	ICD codes from hospital discharge	Cardiovascular mortality by ICD codes from death registry	
Nefrona	• Fatal CHD, MI, fatal, ischemic and hemorrhagic stroke by ICD codes from referring physicians		Cardiovascular mortality by ICD codes from death registry	
OLDW	• CHD: MI by ICD codes from hospital discharge<br>• Stroke: ischemic and hemorrhagic stroke by ICD codes from hospital discharge	ICD codes from hospital discharge	Cardiovascular mortality by ICD codes from hospital discharge and died within 30 days	Medicare/Medicaid claims has linkage and commercial claims has hospital discharge
PSP-CKD	primary care-based codes (Read, predecessor to SNOMED) with linked secondary care outcome data			

Cohorts	Atherosclerotic cardiovascular disease	Heart Failure	Cardiovascular mortality	All-cause mortality
Rancho Bernardo	• CHD: fatal MI by ICD codes from death registry; MI by self-report<br>• Stroke: fatal stroke by ICD codes from death registry; ischemic and hemorrhagic stroke by self-report	Self-report and fatal events using ICD codes		Linkage to death registry
RCAV	• MI, ischemic and hemorrhagic stroke by ICD codes from hospital discharge	ICD codes from hospital discharge	Cardiovascular mortality by ICD codes from hospital discharge and died within 30 days	
REGARDS	• CHD: fatal MI and MI adjudicated by a physician panel<br>• Stroke: fatal, ischemic and hemorrhagic stroke adjudicated by a physician panel	Heart failure events and heart failure deaths were adjudicated by 2 independent clinical investigators who reviewed hospitalization records, and any differences were resolved by discussion.	indication of cardiovascular disease from cause of death adjudicated by a physician panel	Active
SEED				
SKS	• Myocardial infarction, acute coronary syndrome, heart attack, coronary event, stroke, cerebrovascular accident, cerebrovascular event, cerebrovascular disease, as well as indication of cardiovascular disease from cause of death adjudicated by a physician panel			
SMART	• Fatal CHD, MI, fatal, ischemic and hemorrhagic stroke, as well MI, cerebral infarction, cerebral haemorrhage, stroke of undetermined type, certain sudden cardiac death, probable sudden cardiac death, terminal decompensatio cordis, rupture aneurysma abdominal aorta and other cardiovascular disease from cause of death adjudicated by a physician panel			
Sunnybrook				

Cohorts	Atherosclerotic cardiovascular disease	Heart Failure	Cardiovascular mortality	All-cause mortality
ULSAM	• Fatal CHD and MI by ICD codes<br>• Fatal, ischemic and hemorrhagic stroke by ICD-9 codes 430-432, 434 and ICD-10 codes I60-I63		Cardiovascular mortality by ICD codes from death registry	
**Trials**				
ADVANCE	• Fatal CHD, MI, fatal, ischemic and hemorrhagic stroke by ICD codes		Cardiovascular mortality by ICD codes from death registry	
ALTITUDE				
CANVAS Program	All cardiovascular outcomes adjudicated by blinded expert committees	All cardiovascular outcomes adjudicated by blinded expert committees	All cardiovascular outcomes adjudicated by blinded expert committees	Cause-specific mortality adjudicated by blinded expert committees
CARMELINA				
CAROLINA				
CREDENCE	All cardiovascular outcomes adjudicated by a blinded clinical events committee	All cardiovascular outcomes adjudicated by blinded clinical events committee	All cardiovascular outcomes adjudicated by blinded clinical events committee	Cause-specific mortality adjudicated by blinded clinical events committee
DAPA-CKD				
EMPA-KIDNEY	Adjudicated cardiovascular outcomes ascertained from ~6 monthly direct follow-up visits.			
FIDELITY	The cardiovascular outcome was a composite of death from cardiovascular causes, nonfatal myocardial infarction, nonfatal stroke, or hospitalization for heart failure. All outcome events were reviewed and adjudicated by a clinical event committee.			
LEADER				
ONTARGET				
PIONEER™ 6				

Cohorts	Atherosclerotic cardiovascular disease	Heart Failure	Cardiovascular mortality	All-cause mortality
RENAAL	• MI and stroke as well as indication of cardiovascular disease from cause of death adjudicated by a physician panel			
SHARP	Adjudicated cardiovascular outcomes ascertained from ~6 monthly direct follow-up visits.			
SONAR				
SPRINT				
SUSTAIN™ 6				
TRANSCEND				

Listing of Tables and Figures in Excel

Supplementary Table 1. Baseline characteristics including mean CVD risk and number of events in individual cohorts and trials, organized by region and cohort type, included in PREVENT evaluation

Supplementary Table 2. Baseline characteristics of included cohorts and trials for the SCORE2 evaluation, stratified by region and type of study.

Supplementary Table 3. Baseline characteristics including mean CVD risk and number of events in individual cohorts and trials, organized by region and cohort type, included in SCORE2 evaluation

Supplementary Table 4. C-statistics and calibration slopes by individual cohort and trial, organized by region and cohort type ORDERED BY PREVENT

Supplementary Table 5. Calibration slopes and c-statistics of PREVENT model with albuminuria (A) and with glycated haemoglobin (B) by individual cohort and trial, organized by region and cohort type.

Supplementary Table 6. Acronyms or abbreviations for cohorts included in the current study and their key references linked to the Web references.

Supplementary Table 7. Acknowledgements and funding for collaborating cohorts

Supplementary Figure 1. Distribution of 10-year PREVENT ASCVD and HF predicted risks, by study, stratified by validation status and geographical location.

Supplementary Figure 2. Scatter of c-statistic vs mean risk and model-based C-statistic (the maximum discrimination expected given the cohort's case-mix) vs. mean risk

Supplementary Figure 3. Scatter of c-statistic by sex, and scatter of calibration slope by sex. PREVENT total CVD: N = 56 studies; SCORE2 CVD: N = 59 studies; PREVENT ASCVD: N = 61 studies; PREVENT HF: N = 51 studies

Supplementary Figure 4. C-statistics (A) and calibration slopes (B,C) of PREVENT in clinical trials, overall and according to randomised treatment allocation (A.B) or geographical region of the trial.

Supplementary Figure 5. Comparison of C-statistics and calibration slope between PREVENT ASCVD and PCE by individual study; N = 62 Studies

Supplementary Figure 6. Calibration plots of PREVENT base model and PCE by individual cohort and trial, organized by baseline risk and region

References

1. Matsushita, K., *et al.* Association of estimated glomerular filtration rate and albuminuria with all-cause and cardiovascular mortality in general population cohorts: a collaborative meta-analysis. *Lancet* **375**, 2073-2081 (2010).
2. Matsushita, K., *et al.* Estimated glomerular filtration rate and albuminuria for prediction of cardiovascular outcomes: a collaborative meta-analysis of individual participant data. *Lancet Diabetes Endocrinol* **3**, 514-525 (2015).
3. Levey, A.S., *et al.* Expressing the Modification of Diet in Renal Disease Study Equation for Estimating Glomerular Filtration Rate with Standardized Serum Creatinine Values. *Clin. Chem.* **53**, 766-772 (2007).
4. Matsushita, K., *et al.* Comparison of risk prediction using the CKD-EPI equation and the MDRD study equation for estimated glomerular filtration rate. *JAMA* **307**, 1941-1951 (2012).
5. Inker, L.A., *et al.* New Creatinine- and Cystatin C-Based Equations to Estimate GFR without Race. *N Engl J Med* **385**, 1737-1749 (2021).
6. Kidney Disease: Improving Global Outcomes (KDIGO) CKD Work Group. KDIGO 2012 Clinical Practice Guideline for the Evaluation and Management of Chronic Kidney Disease. *Kidney inter., Suppl* **3**, 1-150 (2013).
7. Optum Labs. Optum Labs and OptumLabs Data Warehouse (OLDW) Descriptions and Citation. Vol. PDF. Reproduced with permission from OptumLabs. (n.p., Eden Prairie, MN, March 2023).
