# Supplementary material for: Multinational validation of the PREVENT and SCORE2 cardiovascular risk equations across 6.4 million individuals
Source: Nat Med. 2026 May 5;32(7):2629–38. doi: 10.1038/s41591-026-04437-z (PMC13375529; doi:10.1038/s41591-026-04437-z)
Supplement: Supplementary file 2 [file 41591_2026_4437_MOESM2_ESM.pdf]

nature portfolio

Corresponding author(s): Morgan Grams, Josef Coresh

Last updated by author(s): 04/29/2026

Reporting Summary

Nature Portfolio wishes to improve the reproducibility of the work that we publish. This form provides structure for consistency and transparency in reporting. For further information on Nature Portfolio policies, see our Editorial Policies and the Editorial Policy Checklist.

Statistics

For all statistical analyses, confirm that the following items are present in the figure legend, table legend, main text, or Methods section.

n/a	Confirmed	
☐	☒	The exact sample size (*n*) for each experimental group/condition, given as a discrete number and unit of measurement
☒	☐	A statement on whether measurements were taken from distinct samples or whether the same sample was measured repeatedly
☐	☒	The statistical test(s) used AND whether they are one- or two-sided<br>*Only common tests should be described solely by name; describe more complex techniques in the Methods section.*
☐	☒	A description of all covariates tested
☐	☒	A description of any assumptions or corrections, such as tests of normality and adjustment for multiple comparisons
☐	☒	A full description of the statistical parameters including central tendency (e.g. means) or other basic estimates (e.g. regression coefficient) AND variation (e.g. standard deviation) or associated estimates of uncertainty (e.g. confidence intervals)
☐	☒	For null hypothesis testing, the test statistic (e.g. *F*, *t*, *r*) with confidence intervals, effect sizes, degrees of freedom and *P* value noted<br>*Give P values as exact values whenever suitable.*
☒	☐	For Bayesian analysis, information on the choice of priors and Markov chain Monte Carlo settings
☐	☒	For hierarchical and complex designs, identification of the appropriate level for tests and full reporting of outcomes
☐	☒	Estimates of effect sizes (e.g. Cohen's *d*, Pearson's *r*), indicating how they were calculated

Our web collection on statistics for biologists contains articles on many of the points above.

Software and code

Policy information about availability of computer code

Data collection	Data was collected by each of the individual studies and no code was used for data collection.
Data analysis	All statistical analyses were performed in Stata 18.0. The CKD-PC creates code for analyses centrally and runs analyses on limited datasets sent to the Data Coordinating Center or sends out tailored code to studies unable to send limited datasets, which they then run locally and send the Data Coordinating Center

For manuscripts utilizing custom algorithms or software that are central to the research but not yet described in published literature, software must be made available to editors and reviewers. We strongly encourage code deposition in a community repository (e.g. GitHub). See the Nature Portfolio guidelines for submitting code & software for further information.

Data

Policy information about availability of data

All manuscripts must include a data availability statement. This statement should provide the following information, where applicable:

- Accession codes, unique identifiers, or web links for publicly available datasets
- A description of any restrictions on data availability
- For clinical datasets or third party data, please ensure that the statement adheres to our policy

Under agreement with the participating studies, CKD-PC cannot share individual data with third parties. Inquiries regarding specific analyses should be made to

ckdpc@nyulangone.org and will be responded to in a timely fashion. Investigators may approach the original studies regarding their own policies for data sharing (e.g., https://aric.cscc.unc.edu/aric9/researchers/Obtain_Submit_Data for the Atherosclerosis Risk in Communities Study).

Research involving human participants, their data, or biological material

Policy information about studies with human participants or human data. See also policy information about sex, gender (identity/presentation), and sexual orientation and race, ethnicity and racism.

Reporting on sex and gender	The original PREVENT(TM) equation is stratified by sex due to differences in risk of cardiovascular outcomes.
Reporting on race, ethnicity, or other socially relevant groupings	We did not report on race or ethnicity.
Population characteristics	Individuals in the analyses were aged 30-79 years for PREVENT and 40-89 years for SCORE2, without established CVD. They had to have data on demographics, clinical and laboratory data, and follow-up for atherosclerotic CVD and heart failure.
Recruitment	The studies included in this individual participant level meta-analysis were part of the CKD Prognosis Consortium (CKD-PC). We retained cohorts previously used for validation ("original validation"), and incorporated new cohorts and trials not previously included ("new validation"). Each study needed to have data on participants free of CVD, the key covariates needed for the PREVENT equation, and follow-up for the total CVD outcome.
Ethics oversight	The Institutional Review Board at New York University Grossman School of Medicine waived the need for informed consent for this secondary data analysis and approved this study.

Note that full information on the approval of the study protocol must also be provided in the manuscript.

Field-specific reporting

Please select the one below that is the best fit for your research. If you are not sure, read the appropriate sections before making your selection.

☒ Life sciences ☐ Behavioural & social sciences ☐ Ecological, evolutionary & environmental sciences

For a reference copy of the document with all sections, see nature.com/documents/nr-reporting-summary-flat.pdf

Life sciences study design

All studies must disclose on these points even when the disclosure is negative.

Sample size	Individuals in the analyses were aged 30-79 years for PREVENT and 40-89 years for SCORE2 without established CVD. They had to have data on demographics, clinical and laboratory data, and follow-up for atherosclerotic CVD and heart failure.
Data exclusions	The studies included in this individual participant level meta-analysis were part of the CKD Prognosis Consortium (CKD-PC). We retained cohorts previously used for validation ("original validation"), and incorporated new cohorts and trials not previously included ("new validation"). Each study needed to have data on participants free of CVD, the key covariates needed for the PREVENT equation, and follow-up for the total CVD outcome.
Replication	The performance of the existing PREVENT and SCORE2 equations was replicated in new cohorts and trials not previously included. All analyses were stratified by geographical region (North America, Europe, Asia/other regions, or multi-regional trials).
Randomization	No randomization. Covariates were incorporated as specified in the original equations.
Blinding	N/A

Reporting for specific materials, systems and methods

We require information from authors about some types of materials, experimental systems and methods used in many studies. Here, indicate whether each material, system or method listed is relevant to your study. If you are not sure if a list item applies to your research, read the appropriate section before selecting a response.

Materials & experimental systems

n/a	Involved in the study
☒	☐ Antibodies
☒	☐ Eukaryotic cell lines
☒	☐ Palaeontology and archaeology
☒	☐ Animals and other organisms
☒	☐ Clinical data
☒	☐ Dual use research of concern
☒	☐ Plants

Methods

n/a	Involved in the study
☒	☐ ChIP-seq
☒	☐ Flow cytometry
☒	☐ MRI-based neuroimaging

Plants

Seed stocks	*Report on the source of all seed stocks or other plant material used. If applicable, state the seed stock centre and catalogue number. If plant specimens were collected from the field, describe the collection location, date and sampling procedures.*
Novel plant genotypes	*Describe the methods by which all novel plant genotypes were produced. This includes those generated by transgenic approaches, gene editing, chemical/radiation-based mutagenesis and hybridization. For transgenic lines, describe the transformation method, the number of independent lines analyzed and the generation upon which experiments were performed. For gene-edited lines, describe the editor used, the endogenous sequence targeted for editing, the targeting guide RNA sequence (if applicable) and how the editor was applied.*
Authentication	*Describe any authentication procedures for each seed stock used or novel genotype generated. Describe any experiments used to assess the effect of a mutation and, where applicable, how potential secondary effects (e.g. second site T-DNA insertions, mosiacism, off-target gene editing) were examined.*
